# A new opening for wheat seed production

**DOI:** 10.1093/jxb/erx430

**Published:** 2018-01-23

**Authors:** Laura E Dixon, Stefano Bencivenga, Scott A Boden

**Affiliations:** Department of Crop Genetics, John Innes Centre, Norwich Research Park, Norwich, United Kingdom

**Keywords:** Fertilization, flower, ovary, pericarp, pollination, wheat

## Abstract

This article comments on:

Okada T, Jayasinghe R, Nansamba M, Baes M, Warner P, Kouidri A, Correia D, Nguyen V, Whitford R, Baumann U. 2017. Unfertilized ovary pushes wheat flower open for cross-pollination. Journal of Experimental Botany 69, 399–412.

**Crop plant domestication has targeted a variety of traits, including synchronous development of ovules and stamens to maximize fertilization and seed production. In wheat, with its autogamous, or self-fertilizing, flowers, this is very attractive for guaranteeing yield but extremely frustrating for a researcher trying to cross individuals of distinct genotypes, and even more so for a breeder trying to generate hybrids. Now, Okada *et al.* (2017) have provided a turning point by characterizing the developmental physiology of wheat florets opening after a few days post-anthesis (‘second opening’). This additional opportunity for pollination facilitates out-crossing, and provides a method to further understand the regulation of wheat flower architecture and development.**

We are currently facing a global challenge to sustainably increase crop yields, with the projected requirements far exceeding current production. To meet these needs, a step change in yield production similar to that observed during the Green Revolution is required. One method that has been postulated to provide such a change involves the successful implementation of hybrid wheat production. If hybrid wheat follows the same biology observed in other crops, such as hybrid maize and rice (Tester and Langridge, 2010), it is anticipated to produce a significant yield increase along with other vigour-related benefits, such as disease resistance and increased robustness to abiotic stresses including drought and temperature fluctuations (Tester and Langridge, 2010; Longin *et al.*, 2012). Successful development of hybrid wheat to produce superior yielding and high-quality cultivars could also facilitate an economically sustainable expansion of wheat cultivation into regions that are currently difficult to regulate for commercial breeding companies.

## Towards out-crossing wheat

The major limitation in the production of hybrid wheat is similar to that faced by researchers crossing diverse genotypes under glasshouse conditions: the autogamous, or self-fertilizing, nature of the wheat flower. Often when the anthers emerge, they have already dehisced and the flower has self-pollinated. Currently, self-fertilization is prevented in hybrid wheat production by two methods (Whitford *et al.*, 2013; Mette *et al.*, 2015). The first is chemical application to the female receptor lines. This is expensive, can give variable results and requires precision spraying in favourable weather conditions to avoid triggering sterility in the male donor lines, which are grown in close proximity (see Box 1).

**Box 1. Hybrid wheat seed production**

(A) A cartoon illustrating the field arrangement of the male pollen donor line and female receptor line commonly used for hybrid seed production. The male pollen donor line is taller than the female receptor line, and extrudes anthers to release pollen from the flowers prior to dehiscence. Pollen is then dispersed over the shorter female receptor line, such as by wind, and received by the male-sterile open florets (yellow star). (B) A schematic illustrating the ‘second opening’ of a male-sterile floret (yellow star), generated genetically or using chemical treatment, relative to a floret with fertile anthers. In florets with fertile anthers, pollen is released from the anthers and received by the stigma; this leads to fertilization of the ovule and initiation of grain development, with vertical growth within the floret. In male-sterile florets, radial swelling of the ovary helps push open the floret, so that pollen from a male donor line can be received by a female receptor line.

**Figure:**
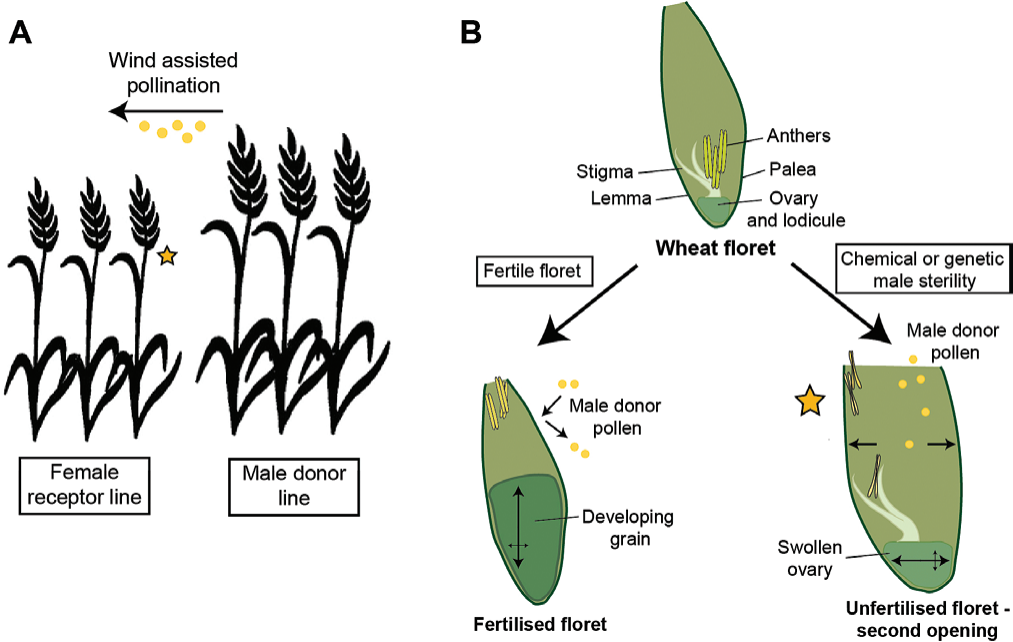

The second method involves using male-sterile lines that do not produce pollen, which is favourable because it removes the need for chemical manipulation. However, use of male-sterile lines alone is insufficient, as they must also be receptive via an open flower to be pollinated by neighbouring wheat plants. It is this aspect of wheat flower development, characterized by the first and second openings of the wheat flower, that has been reported by Okada *et al.* (2017). The authors show that male-sterile lines have a prolonged second opening, facilitated by radial swelling of the unfertilized ovary (see Box 1), which increases the opportunity for cross-pollination. The open flower habit also has some attractive applications in the laboratory – crossing of lines is laborious and prone to failure, as capturing pollen at the correct stage is a lottery. Using lines with an open, receptive female flower would make it possible to pollinate by wind-assisted pollination, dramatically increasing the chance of successful fertilization.

## Further advantages of understanding the wheat flower

Beyond these practical applications, the work of Okada *et al*. also presents new research opportunities. For example, the system provides an interesting way of characterizing the cross-talk between fertilization and ovule development or, more generally, the perception of fertilization by the plant. Fertilization is critical in a plant’s life-cycle and more generally for evolution. As already mentioned, ovule development is synchronized with stamen formation to guarantee successful fertilization in many self-pollinating plant species. When fertilization is unsuccessful, the plants respond by increasing the chances of new fertilization events in ways that depend on plant type. For example, in the model plant Arabidopsis, flower production and lifetime are strictly dependent on fertilization, with an absence of fertilization causing sustained production of flowers and a delay in senescence (Wuest *et al.*, 2016). While some of these responses are shared by wheat (e.g. delayed senescence), wheat displays an intimate connection between fertilization and floret development by modifying the programmed cell death of the mesocarp (Okada *et al.*, 2017).

Furthermore, the paper highlights an interesting evolutionary perspective regarding how biological functions can be co-opted – accumulation of nutrients in the pericarp for embryogenesis/seed development is being redirected for a different function, specifically the opening of the flower. Understanding the efficiency of this process will require deeper investigation to determine whether plants with a more open flower are also the ones that cross-fertilize more frequently, and the effect of the delay in fertilization on seed vigour. If more-open flowers and more-frequent cross-fertilization do correlate it would suggest a close link between second opening and cross-pollination and provide new evolutionary insights. For example, it would be interesting to repeat the ovary analysis in Okada *et al.* (2017) using wheat populations with various degrees of out-crossing (Martin, 1990; Hucl, 1996), and to investigate whether the success of out-crossing is associated with particular environmental conditions to determine if cross-pollination, or the alternative auto-pollination, provides an advantage under certain growth conditions or geographical regions.

An exciting next step will be to investigate the possibility of uncoupling seed development from fertilization. This has already been achieved in Arabidopsis (Chaudury *et al.*, 1997) where in the mutant *fis* the seeds develop without fertilization. Using the analysis in Okada *et al.* (2017), a mutagenesis of male-sterile plants and then screening for spikes that produce ovaries with a phenotype resembling fertilized seeds would provide the genetic material required to dissect this response.

Okada *et al.* (2017) have conducted a rigorous and elegant analysis which can be used both to enhance the development of hybrid crops and to further fundamental understanding of the mechanisms involved in fertilization and seed development. This knowledge can also be applied to different plant species to better follow the mechanisms controlling seed production under changeable environments, which will be a continuing trait of importance for sustainable grain production of our most important crops.
